# FT-Raman and FTIR spectroscopy as a tools showing marker of platinum-resistant phenomena in women suffering from ovarian cancer

**DOI:** 10.1038/s41598-024-61775-z

**Published:** 2024-05-14

**Authors:** Marta Kluz-Barłowska, Tomasz Kluz, Wiesław Paja, Krzysztof Pancerz, Monika Łączyńska-Madera, Paulina Miziak, Jozef Cebulski, Joanna Depciuch

**Affiliations:** 1 Department of Pathology, Fryderyk Chopin University Hospital, F. Szopena 2, 35-055 Rzeszow, Poland; 2 Department of Gynecology, Gynecology Oncology and Obstetrics, Fryderyk Chopin University Hospital, F. Szopena 2, 35-055 Rzeszow, Poland; 3 Institute of Medical Sciences, Medical College of Rzeszow University, Kopisto 2a, 35-959 Rzeszow, Poland; 4 Institute of Computer Science, College of Natural Sciences, University of Rzeszow, Rzeszow, Poland; 5 Institute of Philosophy, John Paul II Catholic University of Lublin, Lublin, Poland; 6 Department of Biochemistry and Molecular Biology, Medical University of Lublin, 20-093 Lublin, Poland; 7 Institute of Physics, College of Natural Sciences, University of Rzeszow, 35959 Rzeszow, Poland; 8 Institute of Nuclear Physics Polish Academy of Sciences, 31342 Krakow, Poland

**Keywords:** FT-Raman, FTIR, ROC, Multivariate analyses, Platinum-resistant ovarian cancer, Correlation, Infrared spectroscopy, Medical and clinical diagnostics, Cancer, Raman spectroscopy, Diagnostic markers

## Abstract

Platinum-resistant phenomena in ovarian cancer is very dangerous for women suffering from this disease, because reduces the chances of complete recovery. Unfortunately, until now there are no methods to verify whether a woman with ovarian cancer is platinum-resistant. Importantly, histopathology images also were not shown differences in the ovarian cancer between platinum-resistant and platinum-sensitive tissues. Therefore, in this study, Fourier Transform InfraRed (FTIR) and FT-Raman spectroscopy techniques were used to find chemical differences between platinum-resistant and platinum-sensitive ovarian cancer tissues. Furthermore, Principal Component Analysis (PCA) and machine learning methods were performed to show if it possible to differentiate these two kind of tissues as well as to propose spectroscopy marker of platinum-resistant. Indeed, obtained results showed, that in platinum-resistant ovarian cancer tissues higher amount of phospholipids, proteins and lipids were visible, however when the ratio between intensities of peaks at 1637 cm^−1^ (FTIR) and at 2944 cm^−1^ (Raman) and every peaks in spectra was calculated, difference between groups of samples were not noticed. Moreover, structural changes visible as a shift of peaks were noticed for C–O–C, C–H bending and amide II bonds. PCA clearly showed, that PC1 can be used to differentiate platinum-resistant and platinum-sensitive ovarian cancer tissues, while two-trace two-dimensional correlation spectra (2T2D-COS) showed, that only in amide II, amide I and asymmetric CH lipids vibrations correlation between two analyzed types of tissues were noticed. Finally, machine learning algorithms showed, that values of accuracy, sensitivity and specificity were near to 100% for FTIR and around 95% for FT-Raman spectroscopy. Using decision tree peaks at 1777 cm^−1^, 2974 cm^−1^ (FTIR) and 1714 cm^−1^, 2817 cm^−1^ (FT-Raman) were proposed as spectroscopy marker of platinum-resistant.

## Introduction

Ovarian malignancies are heterogenous neoplasms with distinct origin, precursor lesions, clinical course, risk factors, molecular profiles, treatment and outcomes. Histologically, WHO classifies ovarian malignancies in distinct groups based on their origin, the most frequently encountered are epithelial ovarian cancers (EOC), constituting 90% of malignant ovarian tumors^1^.

Data published by GLOBOCAN in 2020 showed that there had been 313,959 new cases and 207,252 deaths due to ovarian cancer, which makes it 8th most commonly diagnosed cancer in females and is ranked 8th as far as cause of cancer deaths is concerned^2^. Interestingly, ovarian carcinoma is the leading cause of death in women diagnosed with gynecological cancers^3,4^. Since, there is no reliable screening program and the course of disease is often asymptomatic, many patients have clinically advanced cancer at the time of diagnosis. The staging is determined by FIGO classification, which is based on both surgical and histopathological assessment. In vast majority of cases the therapy of ovarian cancer cannot be based solely on cytoreductive surgery and systemic treatment is crucial.

Platinum-based compounds are currently in use not only for ovarian cancer, but also lung cancer, head and neck cancer, breast cancer and many others.

Nowadays the 1st line treatment in ovarian carcinoma consists of the following: either cytoreductive surgery or neoadjuvant chemotherapy, addition of bevacizumab to carboplatin and paclitaxel to the protocol and in the case of positive response to platinum-based compounds, implementation of PARP inhibitors^5^. However, up to 25% of women with ovarian cancer have so-called platinum-refractory disease. Even if patients are sensitive to 1st line platinum therapy may develop recurrence and acquire progressive resistance over time^6^. Generally, patients treated for ovarian cancer may be divided into following categories:

- platinum refractory—disease progressing during therapy or within 4 weeks after the last dose
- platinum resistant—disease progressing within 6 months of platinum-based therapy
- partially platinum sensitive—disease progressing between 6 and 12 months
- platinum sensitive—disease progressing with an interval of more than 12 months^7,8^.

Both platinum refractory and platinum resistant groups of patients have poorer prognosis and are perfect candidates for clinical trials. Drugs used in in such cases include topotecan, PLD, gemcitabine and paclitaxel in combination with bevacizumab. Platinum-based compounds target cancer cells by forming adducts/crosslinks with DNA purine bases, with a preference for guanine. These crosslinks result in DNA damage that impedes proper genome replication, transcription, and triggers cell apoptosis^9^. The accumulation of platinum antitumor agents inside the cells is the necessary assurance of cytotoxicity, so decreased influx or increased efflux is responsible for platinum resistance^10^. Unfortunately, until now, methods showed platinum-resistant phenomena at the beginning of therapy process are not existed. Therefore, it is necessary to find technique, which will show whether the patient is platinum resistant. Good candidate can be spectroscopies techniques such as FTIR and Raman, which showing biochemical characterization of measured samples^11,12^. Consequently, also differences between analyzed sample can be detected using these methods^13^. Therefore, in this study, FTIR and FT-Raman spectroscopy were used to determine chemical differences between platinum-resistant and platinum-sensitive woman suffering from ovarian cancer. For this purpose, slides from ovarian cancer tissues were cut and measured using two complementary spectroscopy techniques. Moreover, to show if it possible to differentiate ovarian cancer tissues obtained from platinum-resistant and platinum-sensitive women using FTIR and FT-Raman methods, PCA analysis were done. While to obtain information about accuracy, selectivity and sensitivity of spectroscopy methods, ROC curve were used. Finally, decision tree were calculated to show FTIR and FT-Raman spectroscopy markers of platinum-resistant phenomena in ovarian cancer tissues.

## Results

**Figure 1:**
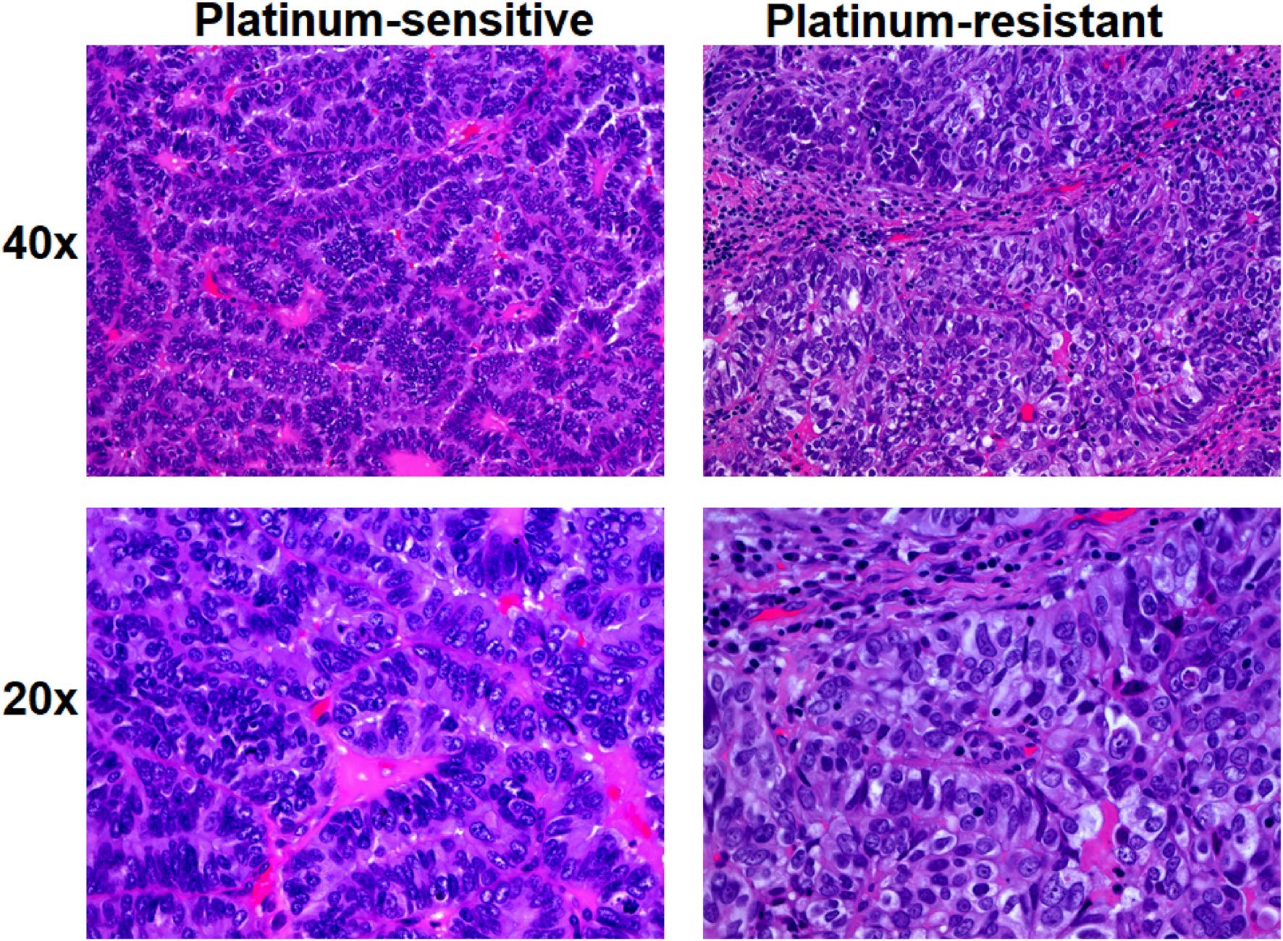
High grade platinum-sensitive (left column) and platinum-resistant (right column) Serous Ovarian Cancer, H&E with 40 × and 20 × magnification.

In this study, FTIR and FT-Raman spectroscopy techniques were proposed as a methods detected differences between platinum-resistant and platinum-sensitive ovarian cancer tissues, which were not differentiate, when currently methods were used, which was visible in Fig. 1.

**Figure 2:**
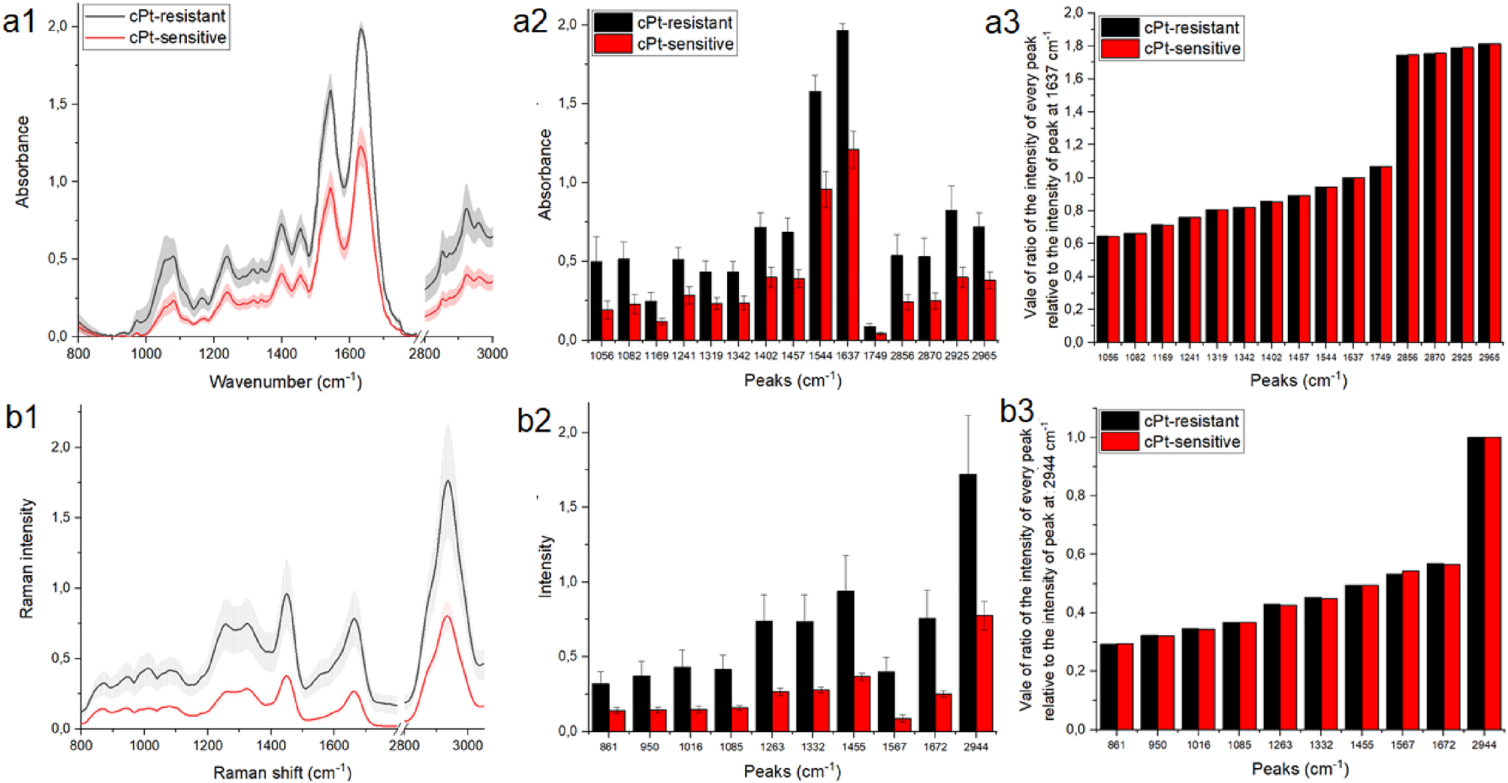
Average of FTIR (**a1**) and FT-Raman (**b1**) spectra ± standard deviation (SD) of ovarian cancer tissues collected from platinum-sensitive (red curve) and platinum-resistant (black color) women; average value of peaks FTIR absorbances (**a2**) and Raman intensities (**b2**) ± SD, where # mean significant differences between platinum-sensitive and platinum-resistant woman. Vale of ratio of the intensity of every peak relative to the intensity of peak at 1637 cm^−1^ for FTIR measurement and at 2944 cm^−1^ for Raman one for FTIR (**a3**) and Raman (**b3**) data. The degree of significance was denoted as p < 0.05.

As was visible in Fig. 1, until now histopathological differences between platinum-resistant and platinum-sensitive tissues were not found. High grade serous ovarian cancer microscopically presets as areas of columnar/cuboidal cells forming solid masses, papillae or slit-like spaces^14^. As depicted there was significant nuclear atypia and pleomorphism, often with bizarre nucleus. Nucleolus was prominent and the mitotic index was high. Apart from that, necrosis was frequent. In immunohistochemistry in presented cases the followed antigens were being expressed: PAX8, WT1, p16, BRCA1, CK7, ER, aberrant p53, high Ki67 index. Both patients had the same result of histopathological examination: high grade serous ovarian cancer and the same stage according to FIGO classification. Yet, only one of them responded to platinum-based therapy. Unfortunately, so far neither histopathological examination, or immunohistochemistry implemented in everyday practice were of any help in determining and predicting reaction to systemic treatment, however interesting studies were being carried out regarding the use of immunohistochemistry in anticipating the reaction to platinum-based compounds^15^. Therefore, in this study we purposed FT-Raman and FTIR spectroscopy to find chemical differences in two analyzed types of ovarian cancer tissues (Fig. 2).

In both types of tissues, the same bonds in FTIR as well in FT-Raman spectrum were noticed.

In FTIR spectra of ovarian tissues (Fig. 2a1), five characteristic ranges were visible: PO^2−^ and C–O–C antisymmetric and symmetric stretching vibrations from phospholipids and esters (1000–1250 cm^−1^), C–H bending vibrations of amino acid side chains and some lipids (1300–1500 cm^−1^)^16,17^, stretching vibrations of C=O, C–N and N–H functional groups in proteins (1500–1700 cm^−1^)^18^, C=O stretching vibrations of phospholipids in the esters (1700–1800 cm^−1^), C−H stretching region of fatty acids (2800–3000 cm^−1^)^19,20^. In first range PO^2−^ symmetric, C–O stretching vibrations and PO^2−^ asymmetric stretching vibrations were placed around 1050 cm^−1^, 1085 cm^−1^ and 1240 cm^−1^, respectively^21^. Moreover C–O–C bonds between the glycerol carbon and fatty acid ester carbon of triglycerides were noticed around 1170 cm^−1^^22^. Functional groups, which were observed in amino acid side chains and some lipids were noticed as C–H bending (around 1410 cm^−1^)^19^ and CH_2_ bending vibrations (around 1450 cm^−1^)^17^. In proteins vibrations, N–H bending in plane and C–N stretching vibrations of amide III were noticed around 1330 cm^−1^, while the same functional groups but originated from amide II were placed at 1544 cm^−1^ (importantly in amide II 40–60% was from N–H, while 18–40% from C–N)^17^. Amide I vibrations were visible around 1640 cm^−1^ corresponding from stretching vibrations of C=O (70%) and stretching vibrations of C–N/bending vibrations of N–H^17^. Esters and lipids vibrations were located at 1740 cm^−1^, 2860 cm^−1^, 2930 cm^−1^ and 2970 cm^−1^ and they were corresponding from C=O stretching vibrations of lipid esters as well as CH_3_ and CH_2_ symmetric and asymmetric stretching vibrations^23^.

In FT-Raman spectra presented in Fig. 2b1 four characteristic ranges originating from respectively chemical compounds were visible^24^. In first range (800–1200 cm^−1^), C–N vibrations of membrane phospholipid head, C–H in-plane bending mode from hydroxyproline and symmetric ring breathing mode of phenylalanine were visible ~ 861 cm^−1^, ~ 950 cm^−1^, ~ 1016 cm^−1^, respectively^24^. Moreover, symmetric phosphate stretching vibrations of DNA/C–N stretching of protein were noticed around 1085 cm^−1^^24^. Second range (1200–1400 cm^−1^) was corresponded from C–N stretching and N–H bending vibrations form amide III (around 1260 cm^−1^) with contributions from CH_3_ wagging and CH_2_ bending vibrations from proteins and lipids (around 1325 cm^−1^ and 1455 cm^−1^, respectively)^24^. In the region between 1700 and 1500 cm^−1^ amide II and C=O stretching vibrations from amide I were visible around 1570 cm^−1^, 1670 cm^−1^^24^, respectively, while in last range, C–H lipids vibrations were observed at 2942 cm^−1^^24,25^.

Figure 2a2,b2 clearly showed, that in ovarian-resistant cancer tissues significant higher value of FTIR absorbances and Raman intensities of all analyzed functional groups were noticed. However, when ratio of the intensity of every peak relative to the intensity of the peaks with the highest intensities was calculated, differences between platinum-resistant and platinum-sensitive was not observed, Fig. 2a3,b3. The detailed positions of the described peaks for each analyzed ovarian tissues were located in Table 1, while schemes of peak splitting for shoulder peaks at 1402 cm^−1^ 1395 cm^−1^, 2925 cm^−1^, and 2930 cm^−1^ (FTIR) and Raman peaks at 1567 cm^−1^ and 1598 cm^−1^ were presented in Supplementary Information S1.

**Table 1:** Peaks analyzed in the FTIR and FT-Raman spectra of platinum-sensitive and platinum-resistant ovarian tissues with a band assigned and differences between positions, where “*” means the shift higher than 4 cm^−1^ for FTIR spectra and 8 cm^−1^ for FT-Raman spectra.

cPt-resistant	cPt-sensitive	Bands
FTIR spectroscopy (cm^−1^)		
1056	1055	Symmetric stretching of PO^2−^ (phospholipids, DNA)^21^
1082	1084	C–O stretching vibrations^21^
1169	1164 *	C–O–C bonds between the glycerol carbon and fatty acid ester carbon^22^
1241	1242	Asymmetric stretching of PO^2−^ (phospholipids, DNA)^21^
1319	1320	N–H bending in plane and C–N stretching of amide III^17^
1342	1342	N–H bending in plane and C–N stretching of amide III^17^
1401	1395 *	C–H bending of amino acid side chains and some lipids^19^
1457	1457	CH_2_ bending vibrations from lipids and proteins^17^
1544	1546	N–H bending in plane (40–60%) and C–N stretching (18–40%) of amide II^17^
1637	1636	C=O (70%) and stretching vibrations of C–N/bending vibrations of N–H of amide I^17^
1749	1749	C=O lipids^23^
2856	2856	Symmetric stretching of CH_3_ from lipids^23^
2870	2871	Asymmetric stretching of CH_2_ from lipids^23^
2925	2927	Asymmetric stretching of CH_3_ from lipids^23^
2965	2965	Symmetric stretching of CH_2_ from lipids^23^
FT-Raman spectroscopy (cm^−1^)		
861	865	C–N vibrations of membrane phospholipid head^24^
950	946	C–H in-plane bending mode from hydroxyproline^24^
1016	1012	Symmetric ring breathing mode of phenylalanine^24^
1085	1081	Symmetric phosphate stretching vibrations of DNA/C–N stretching of protein^24^
1263	1255	C–N stretching and N–H bending vibrations form amide III^24^
1332	1321 *	CH_3_ wagging from proteins and lipids^24^
1455	1456	CH_2_ bending vibrations from proteins and lipids^24^
1567	1598 *	Amide II^24^
1672	1664	C=O stretching vibrations from amide I^24^
2944	2941	CH lipids vibrations^24,25^

Table 1 showed, that in FTIR spectrum, peaks originating from C–O–C bonds between the glycerol carbon and fatty acid ester carbon and C-H bending of amino acid side chains and some lipids were shifted in platinum-resistant ovarian cancer tissues in comparison with platinum-sensitive one. Moreover, for FT-Raman spectrum shifts of peak corresponding to CH_3_ bonds from lipids and proteins towards lower Raman shifts and peak origination from amide II towards higher Raman shifts was observed in platinum-sensitive ovarian cancer tissues.

**Figure 3:**
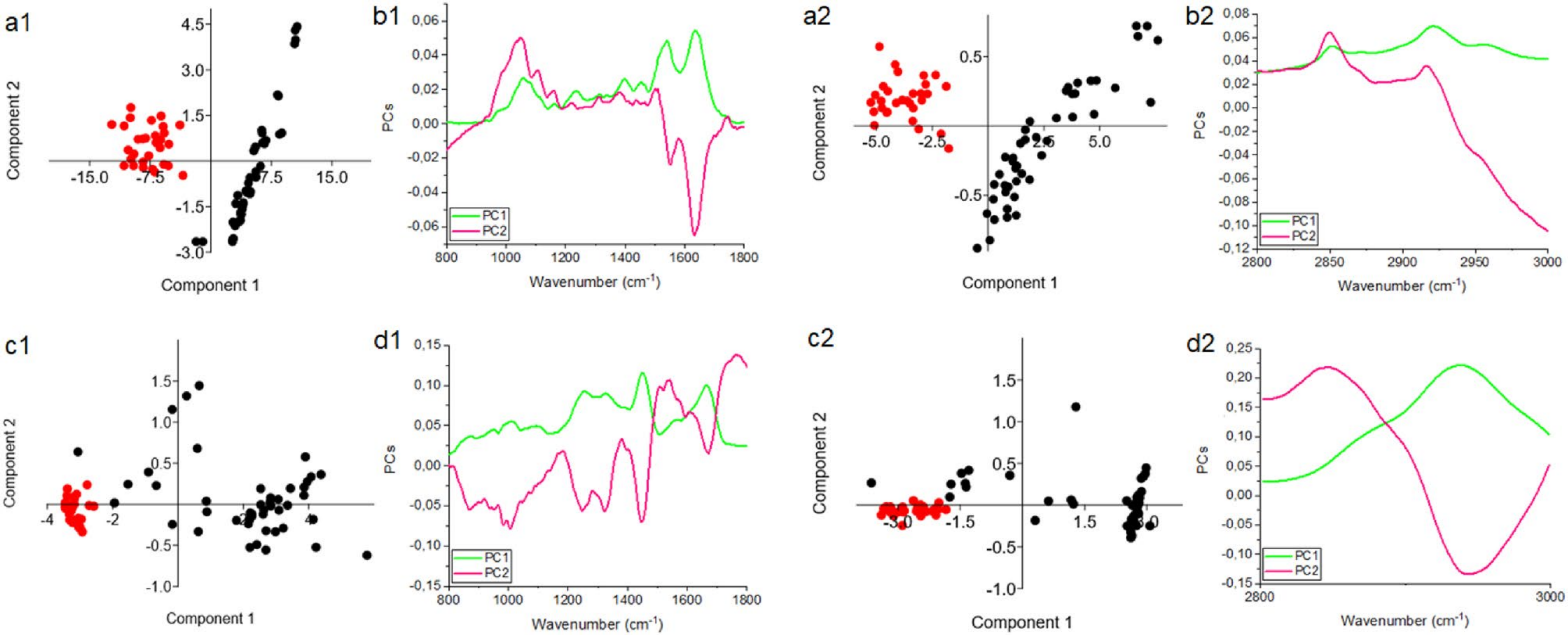
PCA plots (**a1**,**a2**,**c1**,**c2**) with loading plots (**b1**,**b2**,**d1**,**d2**) for FTIR (**a**,**b**) and FT-Raman data (**c**,**d**) in the ranges: 800–1800 cm^−1^ (1) 2800–3000 cm^−1^ (2), where platinum-sensitive samples were marked with red dot and platinum-resistant—black dot.

Principal component analysis was performed to show if possibility of differentiate ovarian cancer tissues collected from platinum-resistant and platinum-sensitive women using FTIR and FT-Raman spectroscopy (Fig. 3). This analysis was done for two spectral ranges: 800–1800 cm^−1^ and 2800–3000 cm^−1^. For both spectroscopy techniques and ranges it was noticed that all samples of ovarian cancer platinum-resistant tissues had positive value of PC1, while platinum-sensitive ovarian cancer tissues—negative. It means that PC1 can be used to distinguish platinum-resistant and while platinum-sensitive ovarian cancer tissues.

**Figure 4:**
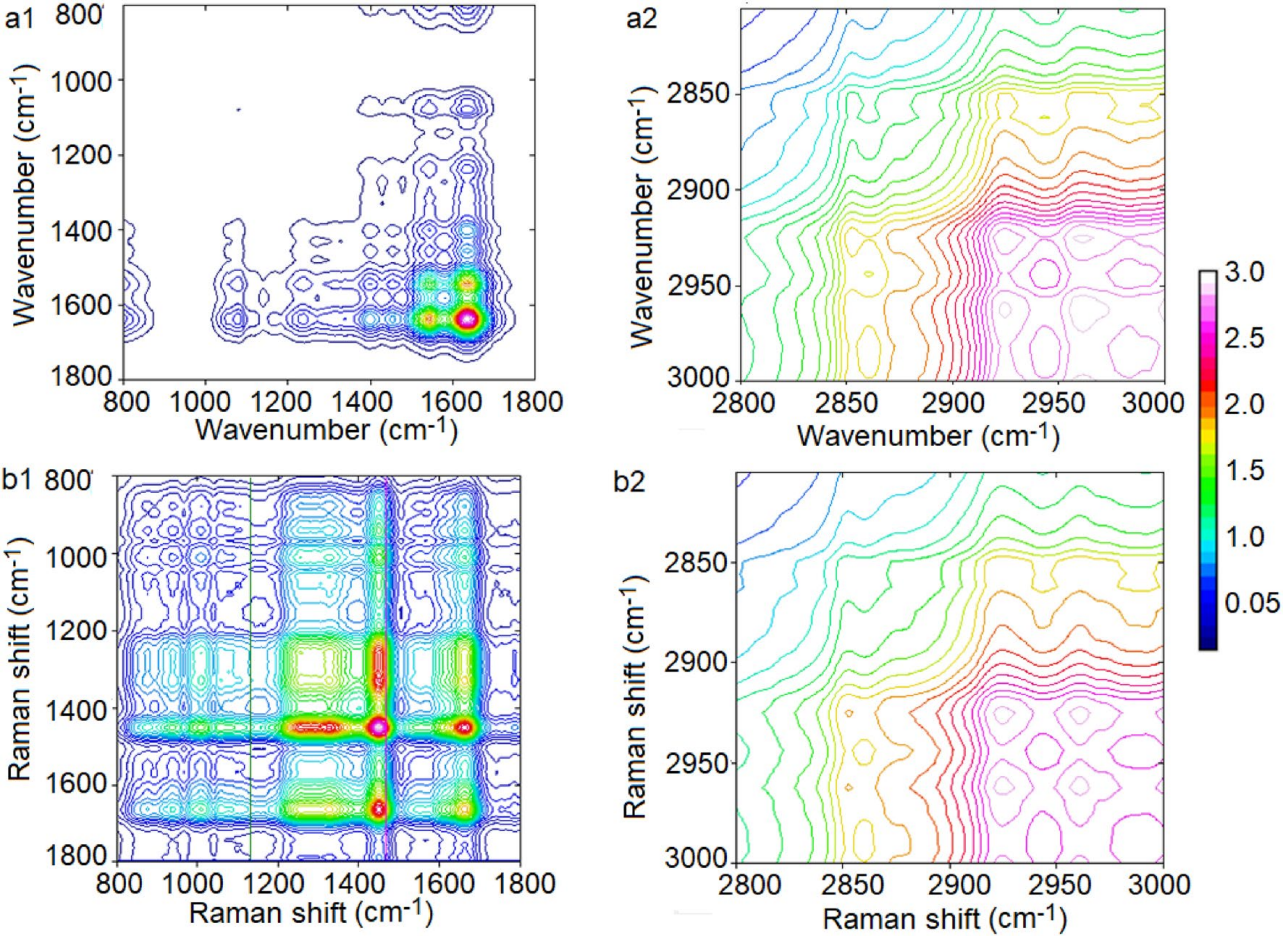
2T2D-COS synchronous 2D plots for FTIR (**a**) and Raman (**b**) ranges: 800–1800 cm^−1^ (1) 2800–3000 cm^−1^ (2), where in x-axis spectrum of platinum-resistant ovarian cancer tissues and in y-axis—platinum-sensitive was located.

Next, to show in which FTIR and FT-Raman ranges spectra collected from platinum-sensitive and platinum-resistant were correlated to each other, 2T2D-COS synchronous was calculated (Fig. 4).

2T2D-COS synchronous 2D plots for FTIR range between 800 and 1800 cm^−1^ showed that both analyzed types of samples were correlated in the range corresponding to amide II and amide I (Fig. 4a1), while in the same range, but in FT-Raman spectra correlation was found in CH_3_, CH_2_ bonds from lipids and proteins, as well as in amide I (Fig. 4b1). It means that higher differences in this range between tissues were noticed in FTIR spectrum. In the range between 2800 and 3000 cm^−1^ correlation was noticed for CH_2_ and CH_3_ asymmetric vibrations in FTIR spectra (Fig. 4a2) and for CH vibrations in FT-Raman spectra (Fig. 4b2.

Principal component analysis showed differentiation between platinum-resistant and platinum-sensitive ovarian cancer tissues for both using spectroscopy techniques. Therefore, using machine learning and ROC curve, accuracy, sensitivity and specificity were calculated, Table 2, while ROC curves for both FTIR and FT-Raman sets were shown in Supplementary Fig. S2.

**Table 2:** Classification results between platinum-resistant and platinum-sensitive ovarian cancer tissues obtained with three different ML algorithms for wavelength ranges of FTIR and FT-Raman spectroscopy.

(cm^−1^)	Algorithm	# of features	Accuracy	Sensitivity	Specificity	Precision	ROC AUC
FTIR 800–3000	SVM	4565	1.00	1.00	1.00	1.00	1.00
	kNN	4565	1.00	1.00	1.00	1.00	1.00
	XGBoost	4565	0.97	0.97	0.98	0.97	0.95
Raman 800–3000	SVM	571	0.97	1.00	0.96	0.94	0.99
	kNN	571	0.97	1.00	0.96	0.94	0.99
	XGBoost	571	0.99	1.00	0.98	0.97	0.98

Table 2 showed, that depending on the using machine learning algorithms, the value of accuracy, sensitivity and specificity was differed for FTIR and FT-Raman measurements. However, it was visible, that vales of calculated parameters were higher for FTIR data. For this technique, accuracy and sensitivity was from 97% (XGBoost) to 100% (SVM, kNN), while specificity was from 98% (XGBoost) to 100% (SVM, kNN). For FT-Raman data, accuracy was 97% (SVM, kNN) or 99% (XGBoost), sensitivity was 100% for all using algorithms, while specificity was from 96% (SVM, kNN) to 100% (XGBoost). Area Under ROC curve values also was depended on the using algorithms and for FTIR data value was 95% for XGBoost and 100% for SVM and kNN. Finally, ROC curve value for FT-Raman data was 98% for XGBoost or 99% for rest two algorithms.

**Figure 5:**
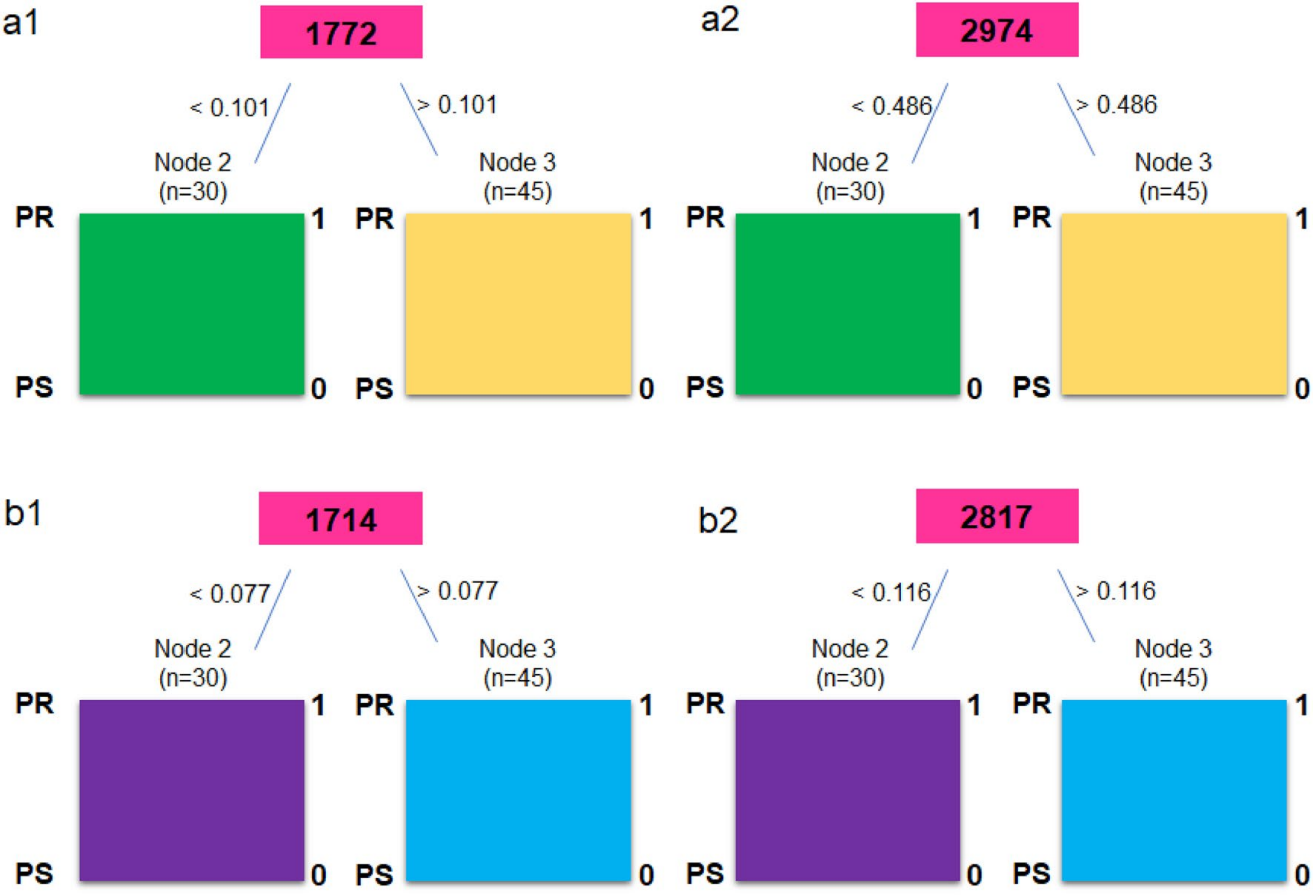
Decision tree for FTIR (**a**) and FT-Raman (**b**) ranges 800–1800 cm^−1^ (1) and 2700–3000 cm^−1^ (2).

Next, to show, which wavenumbers or Raman shifts can be used as a FTIR and FT-Raman markers of platinum-resistant, decision trees were created (Fig. 5).

**Figure 6:**
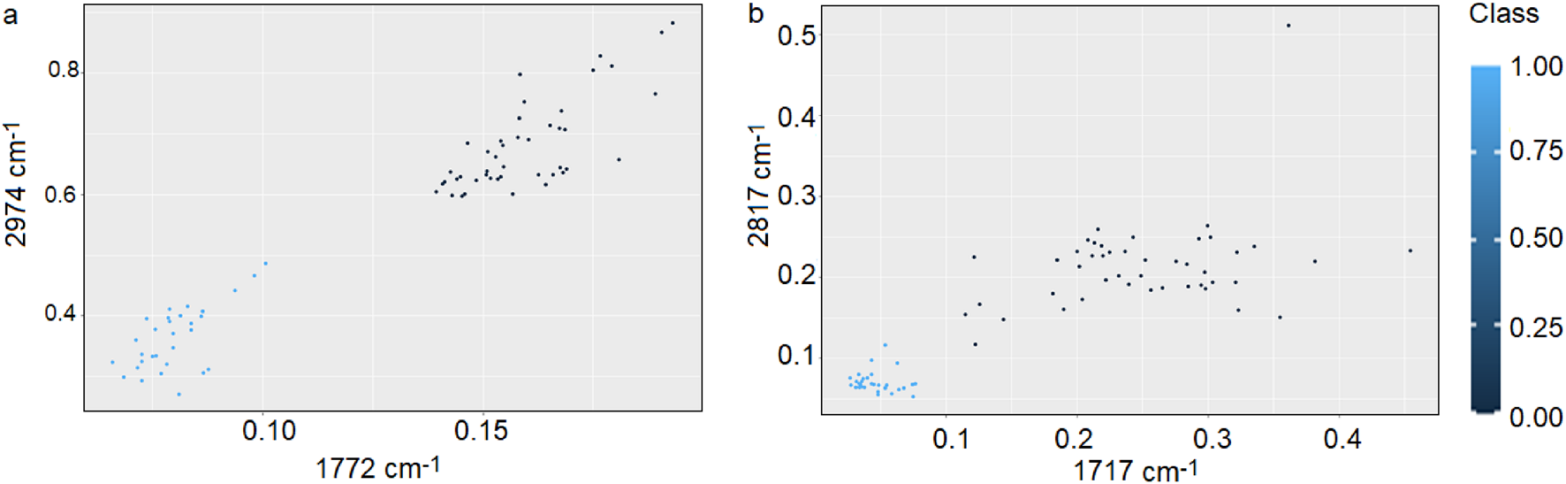
A scatter plot showing two clusters of patients distinguished by two peaks originating from decision tree for FTIR (**a**) and FT-Raman (**b**) data.

Decision tree showed, that FTIR markers of platinum-resistant phenomene in ovarian cancer were at 1777 cm^−1^, 2974 cm^−1^, while FT-Raman markers were placed at 1714 cm^−1^, 2817 cm^−1^. Using these wavenumbers and Raman shifts, cluster analysis was done (Fig. 6).

From scatter plots presented in Fig. 6 it was visible that better clustering for FTIR data was noticed than for FT-Raman one. However, for both using spectroscopy techniques it was observed, that samples collected from ovarian-resistant women had higher value of class than samples collected from women suffering from ovarian cancer but without platinum-resistant phenomena.

## Discussion

The most deadly gynecological cancer is ovarian cancer. The mainstay of treatment for this type of cancer is platinum-based chemotherapy. In the initial phase, most patients are platinum-sensitive. Unfortunately, due to repeated relapses, this cancer is becoming platinum-resistant. Moreover, recurrent recurrence also has a poor prognosis and limited treatment options, the effectiveness of which is also limited. Unfortunately, until now it has not been possible to determine a marker that would clearly show whether a given patient has platinum-resistant ovarian cancer^26^. Therefore, in this study FTIR and FT-Raman spectroscopy techniques were shown as a candidate for diagnostic tool of platinum-resistant ovarian cancer. Taking into account, that resistance to chemotherapeutics based on platinum compounds is a very complex process that affects not only cancer cells, but also the tumor microenvironment^27^, in this study, ovarian cancer tissues were used as a studied materials. Unfortunately, comparison of obtained results with other one, when Authors used FTIR or FT-Raman spectroscopy was not possible, because it is first probe of used these techniques in differentiation platinum-resistant and platinum-sensitive ovarian cancer tissues. Consequently, only comparison with molecular or biochemical results will be presented.

Until now, several types of molecules groups were analyzed as a biomarkers of platinum-resistant phenomena, e.g. glycoprotein biomarkers, liquid biopsy, epigenetic and genetic markers, immune-Related biomarkers and angiogenic markers. All of these markers were so-called circulating biomarkers^28^. In first group (glycoprotein biomarkers), glycoproteins such as CA125 and HE4 were good candidate^29^. In studies where the role of CA125 was analyzed in regulating the sensitivity of epithelial OC cells to various types of genotoxic drugs, it was shown that CA125 inhibits the action of cisplatin and drugs whose main component was cisplatin. More specifically, it was the C-terminal domain (CTD) of CA125 that mediates this inhibition^30^. Human epididymal protein 4 (HE4) was another molecule that may act as a marker of platinum resistance. Studies have shown that in women who do not respond to cisplatin treatment, the serum level 4 (HE4) was higher than in platinum-sensitive women. This level was so high that small concentrations of 4 (HE4) are also recorded in urine^31,32^. In our study, higher level of amides vibrations in FTIR as well as FT-Raman spectra was also noticed (Fig. 2). Moreover, also shifts of amide II in FT-Raman spectra of tissues collected from platinum-resistant woman was visible (Table 1). All of these suggested higher level of proteins and changes in their structure. However, the exact mechanism of inhibition of the action of cisplatin by 4 (HE4) has not been known so far. Angioli et al. showed that serum HE4 levels during first-line chemotherapy predicted the occurrence of platinum-resistant disease in the third cycle of chemotherapy with 100% sensitivity and 85% specificity^33,34^. Moreover, we also noticed, that between both types of tissues correlation was existed in amides vibrations (Fig. 4a1,b1), which also suggested that proteins play important role in platinum-resistant phenomena. On the other hand, higher level of lipids (Fig. 2) in FTIR spectrum of platinum-resistant ovarian cancer tissues was noticed. Taking into account correlation (Supplementary Figs. a2, b2) and that decision trees showed that only peaks originating from lipids vibrations (1777 cm^−1^, 2974 cm^−1^—FTIR 1714 cm^−1^, 2817 cm^−1^—FT-Raman, Fig. 5) can be spectroscopy markers of platinum-resistant marker, we suggested, that lipids could have crucial role in analyzed phenomena. Indeed, it has been shown that the uptake of long-chain fatty acids into cells was very important in the use of cisplatin-based chemotherapy. This uptake was controlled by fatty acid binding protein 4 (FABP4)^35^. Therefore, it can be said that fatty acids may be responsible for bringing cisplatin into cancer cells. However, until now this mechanism is still unknown. Consequently, it is necessary further research. We have shown that ovarian cancer tissues from platinum-sensitive and platinum-resistant patients can be distinguished, Figs. 3, 6. However, realizing that the study sample was not large, the results should be verified on a larger number of respondents.

## Conclusions

In this study we showed that FTIR and FT-Raman spectroscopy techniques combined with PCA can be used for detection chemical differences in ovarian cancer tissues collected from platinum-resistant and platinum-sensitive women. Importantly, accuracy, selectivity and sensitivity of presented methods in our study were around 100% respectively for FTIR and around 95% for FT-Raman measurements. Moreover, in this study it was shown, that correlation between both analyzed types of tissues was visible only in amide II, amide I and asymmetric CH lipids vibrations in FTIR spectra and in CH_3_, C–H bending from lipids and proteins, as well as in amide I and CH lipids bonds in FT-Raman spectra. It means, that in other analyzed vibrations, chemical differences were noticed. Importantly, decision tree showed, that FTIR marker of platinum-resistant phenomena in ovarian cancer was placed at 1777 cm^−1^, 2974 cm^−1^, while FT-Raman marker was placed at 1714 cm^−1^, 2817 cm^−1^.

## Materials and methods

### Materials

All patients, which participated in this study were treated in Fryderyk Chopin University Hospital in Rzeszow between 2017 and 2020. Continuing, tissues from 15 women with average age 52 years with histopathological diagnosis of high-grade ovarian adenocarcinoma, where 9 were platinum-resistant and 6 were platinum-sensitive were obtained during biopsy procedure before chemotherapy in the Fryderyk Chopin University Hospital in Rzeszow. In all patients from which tissues were obtained no mutation, neither in BRCA1 and BRCA 2 gene, or homologous recombination deficiency (HRD) were found based on next-generation sequencing (NGS). Importantly, also histopathological studies were not shown differences between platinum-resistant and platinum-sensitive ovarian tissues. This study was approved by the Bioethics Committee of the Regional Medical Chamber in Rzeszow–24 November 2016 (Resolution No. 90/B/2016). Consequently, all methods were performed in accordance with the relevant guidelines and regulations. Moreover, from all patients informed consent, which was approved by Bioethics Committee of the Regional Medical Chamber in Rzeszow was obtained.

### Methods

After biopsy, part of tissues was frozen in the Eppendorf in temperature − 80 °C. Before measurements, frozen tissues were cut into thin slices, about 10 µm and put on the CaF_2_ slides for FTIR measurements and on the gold substrate dedicated for FT-Raman spectrometer. From each samples, 5 spectra from different location were collected using FTIR and FT-Raman spectrometers. All spectra were normalized using min.-max normalization method. Moreover baseline correction using Rubberband methods and 64 baseline points was applied. Obtained spectra were analyzed using the OPUS 7.0 software. On the other hand, part of materials was used to obtain paraffin block for histopathological analysis.

### Microscope images of tissue stained for histopathological analyses

Cytoreductive procedures, even in the initial stages of the disease, were not pertained to the pelvis only (i.e. hysterectomy, bilateral salpingo-oophorectomy, peritonectomy and pelvic lymphadenectomy) but extended to abdomen (i.e. omentectomy, para-aortic lymphadenectomy). Frequently, in more advanced cases the scope of the surgery may be extended by bowel, liver or pancreas resection.

The tissues removed during the operation are fixed with 10% neutral buffered formalin solution, which pH is ranging from 7.2 to 7.4. The storage temperature was between 20 and 25 °C. The materials obtained during cytoreductive procedures were placed in disposable, special-purpose containers which were adequate for biological material and were resistant to the fixative. It was important to adjust the size and the opening of the container to the stored material, to avoid tissue deformation. Also, the volume of the fixative should be tenfold greater than the size of the stored material. It was essential to label the container properly (i.e. patient’s ID and kind of the material) and these data had to be compatible with the referral. The referral, in terms of ovarian cancer, should contain the following information about the patient: radiotherapy, chemotherapy or hormonotherapy, previous or co-existing malignant neoplasm, familiar BRCA1/2 gene mutation and familiar history of ovarian and breast cancer and previous gynecological operations. Once the material was delivered to the pathology department, the time of fixation had to be strictly observed—small tissue samples had to be grossed from 6 to 48 h since the beginning of fixation, whereas large specimens (organ or its parts) had remain in the fixative from 24 up to 72 h maximally^36^. It should also be noted that all specimens were checked when accepted to the pathology department (refilled with the fixative, cut—pretreatment). The duration of the fixation process was of a great importance as far as immunohistochemistry staining and genetic studies were concerned. When the specimen was fixated grossing examination was performed. Grossing consists of thorough measuring of the specimen, describing the organ and there was macroscopic infiltration of the neoplasm (surface of the ovary, fallopian tubes, serosa of the uterus or other organs) also noting if the surface of the ovary was intact. Afterwards it was necessary to ink the external surface of the ovary and cut the organ along the greatest dimension, including the hilum and detail the cross section. If present, cysts either on the outer surface or on the inner surface of the ovary were characterized (its content, size, inner surface, if uni- or multiloculated, thickness of the cyst wall), with exophytic or papillary structures requiring the most attention when present (if singular the whole had to be sampled, if multiplicitous numerous sections had to be sampled). If solid areas occur, one should measure it, described and gave the percentage of necrosis.

For the microscopic examination it was mandatory to sample 1 section for every 2 cm of the tumor, especially from the solid areas. Each section should not be thicker than 4 mm, while 3 mm was the ideal thickness of the sample, which was later placed in histopathological cassette signed with patient’s ID. The cassettes were placed in tissue processor and subsequently undergo the following processes: fixation (10%neutral buffered formalin), dehydration (ethanol, isopropyl alcohol), clearing (xylene, toluene), wax infiltration (paraffin). The processed material was embedded in paraffin and then, paraffin blocks were cut on a microtome into thin sections (2.5 microns) and placed on a glass slide. The next step was standard hematoxylin–eosin staining and applying of the covering glass. When the microscopic slides were prepared the entire case was undergoing quality-check. Every step of the preparation process was strictly documented. Finally, once the case was assessed by the clinical pathologist, the result of histopathological examination should include the following: histological type according to WHO classification, grading and staging (in coherence with TNM AJCC/UICC—the extent of invasion of the reproductive as well as other organs), vascular invasion and other pathological findings. Based on the result of histopathological examination and surgical assessment the adequate treatment was applied. Histopatological images were taken using Nikon Eclipse Ei with Digital Sight 1000 camera.

### FT-Raman and FTIR measurements

Nicolet NXR 9650 FT-Raman spectrometer (Thermo Fisher Scientific, USA) was used in this study to collect spectra of frozen tissues of ovarian cancer tissues. This spectrometer has Nd:YAG laser with 1064 nm wavelength and a germanium detector. In this study unfocused laser beam was used with a diameter of approximately 100 μm. Moreover, each sample was scanned 64 times with spectral resolution of 8 cm^−1^ and laser power of 1 W. The measurement range was between 150 and 3700 cm^−1^. In the other hand, a Bruker Vertex 70v spectrometer equipped with an attenuated total reflection (ATR) diamond crystal plate was used to collect FTIR spectra of ovarian tissues. The absorbance spectra were collected in the range between 400 and 4000 cm^−1^ using 32 scans and spectral resolution 4 cm^−1^. Before FTIR measurement, a background spectrum was collected. Moreover after each measurement, diamond crystal was cleaned using 70% of ethanol. For measurements, frozen ovarian cancer tissues were cut into thin (10 µm) slides and put on the gold standard for Raman and CaF_2_ glasses for FTIR spectra collected.

### Analyses of FT-Raman and FTIR spectra

To determine statistical significance in the differences of Raman intensities and FTIR absorbances of the amount of lipids, amides and polysaccharides in the spectra of tissues collected from platinum-resistant and platinum-sensitive, one-way ANOVA with Tukey’a post hoc test was done. Significance was defined as less than a 0.05 p-value. Furthermore, to show if FT-Raman and FTIR spectroscopy can be used to differentiate platinum-resistant and platinum-sensitive ovarian tissues, Principal Component Analysis (PCA) was done. For creation PCA plots number of analyses components which showed 95% variances was used. These two analyses were performed using Past 4.10 software. Furthermore to show correlation between platinum-resistant and platinum-sensitive tissues in FT-Raman and FTIR spectra two-trace two-dimensional correlation spectra (2T2D-COS) was used. Moreover, 2T2D-COS was also used to correlate FT-Raman and FTIR spectra of platinum-resistant (i) and platinum-sensitive (ii) to show, which kind of technique showed which characteristic vibrations in analyzed materials. Synchronous spectra gives the characteristics information with good sensitivity. This 2T2D-COS was done using OPUS 7.0 software. PCA and 2T2D-COS were performed for two spectral ranges (800–1800 cm^−1^ and 2800–3000 cm^−1^). Moreover, data obtained from FTIR and FT-Raman and spectroscopy experiments formed the basis for analysis using machine learning methods. Three well-known ML model-building algorithms were used in the analysis: support vector machine (SVM)^37^, Extreme Gradient Boosting (XGBoost)^38^ and k-nearest neighbor classifier (kNN)^39^. The raw experimental data was processed into the form of a so-called decision table. Such an array consists of columns denoting a specific wavelength and rows being a specific patient case. The last column of the array denotes the category, class, of the patient, 0 being a resistant case, 1 being a sensitive case. The quality of classification, diagnosis of patient cases was examined using four parameters: accuracy, sensitivity, specificity and precision and using the area under the ROC curve. A random forest-based feature selection algorithm was used to identify significant wavelength intervals (i.e., in machine learning for selection of significant features)^40^. Based on the analysis of their relevance, it was possible to identify some narrow waveform ranges that were relevant to the identification of sick patient cases. The analysis of relevant wavenumber were visible in Supplementary Fig. S3. In addition, decision tree graphs were obtained for the 800–1800 cm^−1^ and 2800–3000 cm^−1^ for FTIR and FT- Raman data using the C5.0 decision tree construction algorithm. The wavelengths identified in the trees were used to visualize the data as clusters of objects that were similar to each other.

### Supplementary Information

Supplementary Figures.

## Data Availability

The data supporting this study's findings are available from the corresponding author upon reasonable request.
