# Supplementary material for: FT-Raman and FTIR spectroscopy as a tools showing marker of platinum-resistant phenomena in women suffering from ovarian cancer
Source: Sci Rep. 2024 May 14;14:11025. doi: 10.1038/s41598-024-61775-z (PMC11094164; doi:10.1038/s41598-024-61775-z)
Supplement: Supplementary file 1 — Supplementary Figures. [file 41598_2024_61775_MOESM1_ESM.docx]

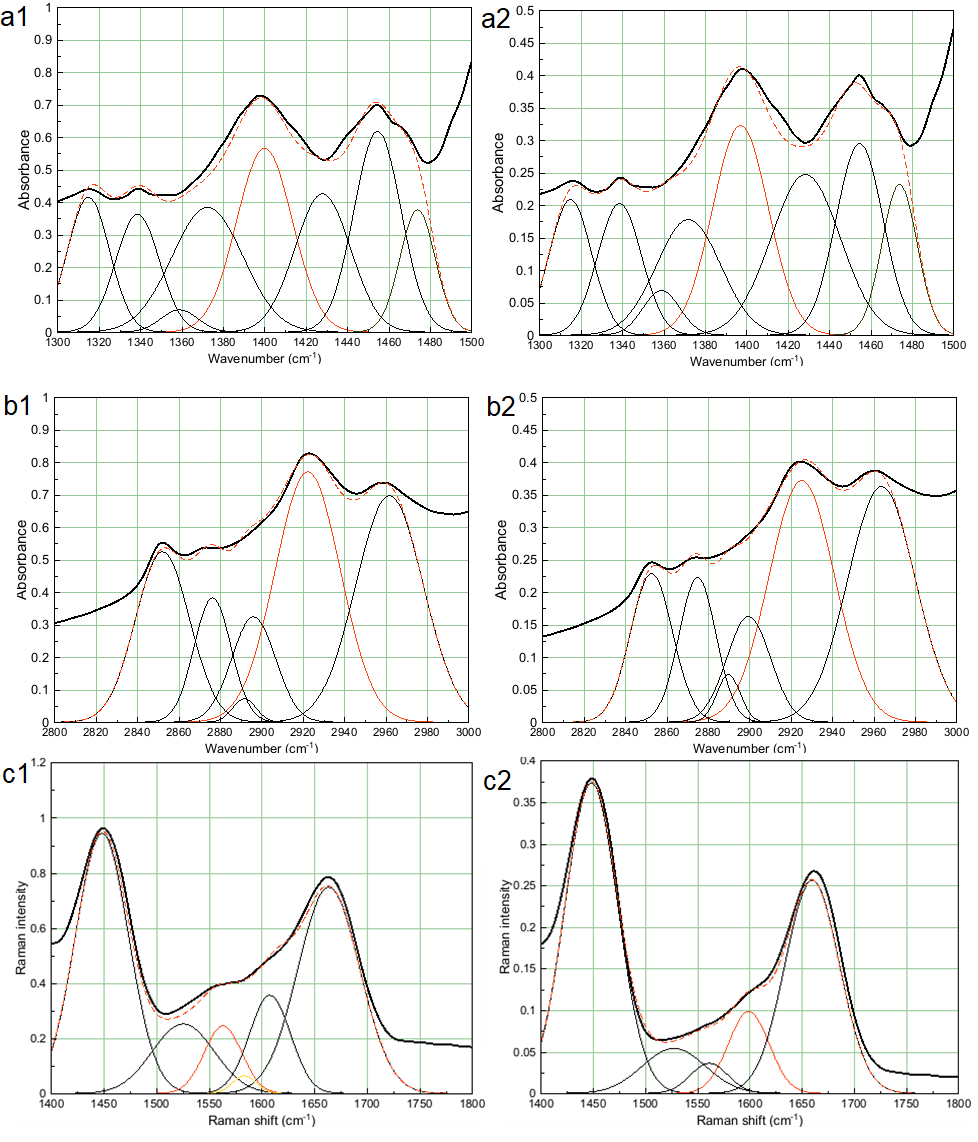


**Figure S1.** Schemes of peak splitting for shoulder peaks at 1401 cm^-1^ (a1) 1395 cm^-1^ (a2), 2925 cm^-^ (b1)^1^, and 2927 cm^-1^ (b2) in FTIR spectra and peaks at 1567 cm^-1^ (c1) and 1598 cm^-1^ (c2) in Raman spectra.


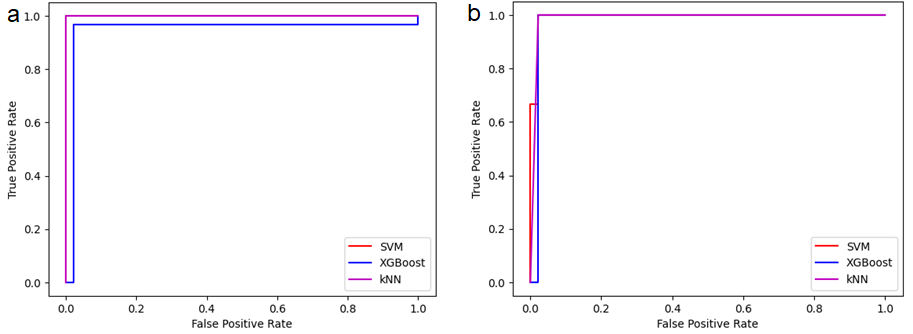


**Figure S2.** ROC curves obtained for individual models (SVM, XGBoost, kNN) for FTIR (a) and FT-Raman.


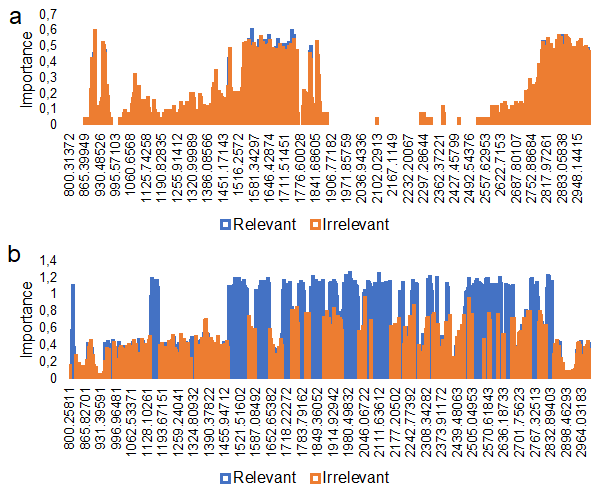


**Figure S3.** Mean importance values of individual wavenumbers obtained with the feature selection algorithm during analysis of the FTIR (a) and FT-Raman (b) data.
